# Increasing Superoxide Production and the Labile Iron Pool in Tumor Cells may Sensitize Them to Extracellular Ascorbate

**DOI:** 10.3389/fonc.2014.00249

**Published:** 2014-09-16

**Authors:** Mark Frederick McCarty, Francisco Contreras

**Affiliations:** ^1^Oasis of Hope Hospital, Tijuana, Mexico

**Keywords:** ascorbate, intravenous, cancer, superoxide, Haber–Weiss, dichloroacetate, ketosis, elesclomol

## Abstract

Low millimolar concentrations of ascorbate are capable of inflicting lethal damage on a high proportion of cancer cells lines, yet leave non-transformed cell lines unscathed. Extracellular generation of hydrogen peroxide, reflecting reduction of molecular oxygen by ascorbate, has been shown to mediate this effect. Although some cancer cell lines express low catalase activity, this cannot fully explain the selective sensitivity of cancer cells to hydrogen peroxide. Ranzato and colleagues have presented evidence for a plausible new explanation of this sensitivity – a high proportion of cancers, via NADPH oxidase complexes or dysfunctional mitochondria, produce elevated amounts of superoxide. This superoxide, via a transition metal-catalyzed transfer of an electron to the hydrogen peroxide produced by ascorbate, can generate deadly hydroxyl radical (Haber–Weiss reaction). It thus can be predicted that concurrent measures which somewhat selectively boost superoxide production in cancers will enhance their sensitivity to i.v. ascorbate therapy. One way to achieve this is to increase the provision of substrate to cancer mitochondria. Measures which inhibit the constitutive hypoxia-inducible factor-1 (HIF-1) activity in cancers (such as salsalate and mTORC1 inhibitors, or an improvement of tumor oxygenation), or that inhibit the HIF-1-inducible pyruvate dehydrogenase kinase (such as dichloroacetate), can be expected to increase pyruvate oxidation. A ketogenic diet should provide more lipid substrate for tumor mitochondria. The cancer-killing activity of 42°C hyperthermia is to some degree contingent on an increase in oxidative stress, likely of mitochondrial origin; reports that hydrogen peroxide synergizes with hyperthermia in killing cancer cells suggest that hyperthermia and i.v. ascorbate could potentiate each other’s efficacy. A concurrent enhancement of tumor oxygenation might improve results by decreasing HIF-1 activity while increasing the interaction of ascorbic acid with oxygen. An increased pool of labile iron in cancer cells may contribute to the selective susceptibility of many cancers to i.v. ascorbate; antagonism of NF-kappaB activity with salicylate, and intravenous iron administration, could be employed to further elevate free iron in cancers.

## Increased Superoxide Production may Rationalize Selective Sensitivity of Cancers to Ascorbate

Low millimolar concentrations of ascorbate – achievable clinically by intravenous high-dose administration, but not oral administration (1, 2) – are capable of killing many cancer cell lines *in vitro*, whereas, non-malignant cell lines are not harmed by ascorbate in concentrations as high as 20 mM (3, 4). In mice bearing transplanted tumors, parenteral administration of high-dose ascorbate has been reported to slow cancer growth, and to potentiate response to concurrent gemcitabine (2, 5, 6).

Extracellular catalase, but not superoxide dismutase, abolishes the toxicity of extracellular ascorbate to cancer cells (3). Levine and colleagues have shown that, *in vivo*, millimolar levels of ascorbate generate superoxide, hydrogen peroxide, and ascorbyl radical in the extracellular space; it is suspected that transition metals bound to proteins in the extracellular space catalyze transfer an electron from ascorbate to molecular oxygen, producing superoxide that is then dismutated to yield hydrogen peroxide. Since superoxide does not readily transit cell membranes, and since extracellular catalase abolishes ascorbate’s toxicity to cancer cells, hydrogen peroxide appears to mediate, in whole or in part, this toxicity. Indeed, Levine’s group has shown that micromolar concentrations of hydrogen peroxide that realistically could be generated by ascorbate kill an ascorbate-sensitive lymphoma cell line in a manner identical to that seen with ascorbate exposure (3). These researchers also demonstrated that exposure of cancer cell lines to dehydroascorbate, in amounts which boosted intracellular ascorbate levels to the same level achieved by extracellular ascorbate exposure, was not toxic to cancer cells; hence, extracellular effects of ascorbate are key to ascorbate’s cytotoxicity (3).

It clearly is of interest to clarify the molecular basis of extracellular ascorbate’s selective toxicity to many cancer cell lines. It has been suggested that the failure of ascorbate to kill non-malignant cells may reflect the superior capacity of these cells to dispose of hydrogen peroxide via catalase or glutathione peroxidase activity. Indeed, there are a number of reports of low catalase activity in various cancer cell lines (7).

However, cancer cells are not always inherently deficient in these activities, and Ranzato et al. reported that, although a malignant mesothelioma cell line was much more sensitive to the lethality of extracellular ascorbate than were normal mesothelial cells, the latter cell line actually had *less* catalase activity than the mesothelioma (8). These researchers then discovered that the mesothelioma cells were generating more superoxide than the normal mesothelial cells – via Nox4 activity, as well as mitochondrial activity – and that measures which suppressed superoxide production in the mesothelioma (apocynin, rotenone, and Nox4 anti-sense) all markedly protected the cancer cells from ascorbate exposure. They therefore postulated that a Haber–Weiss reaction, in which extracellularly generated hydrogen peroxide was interacting with superoxide produced within the cancer cells to generate vicious hydroxyl radicals, was a key mediator of the lethal impact of ascorbate exposure on cancer cells. This model is consistent with a previous report that pretreatment of cancer cells with the cell-permeant iron chelator deferoxamine – but not cell-impermeant iron chelators – protected these cells from subsequent ascorbate exposure (6); iron is of course a catalyst for the Haber–Weiss reaction. The selective sensitivity of many cancer cell lines to killing by ascorbate may therefore reflect, to a large degree, the tendency of cancers to generate superoxide at an increased rate – complemented in some but not all cases by a deficiency of superoxide dismutase and/or peroxidase activities.

Spitz and colleagues have confirmed that superoxide production is increased in a number of cancer cell lines, and demonstrate that much of this superoxide is of mitochondrial origin (9). When cancer cells as well as non-malignant cell lines were exposed to the mitochondrial respiratory inhibitor antimycin, the increase in superoxide production was markedly higher in the cancer cells. This suggests that the mitochondria of cancer cells tend to have dysfunctional respiratory chains, such that a higher proportion of the electrons flowing down these chains are diverted to superoxide production at complexes I, II, and III. Indeed, there are a number of reports that the mitochondria of cancer cells are structurally abnormal, and that mutations of mitochondrial DNA are more common in cancer (10–13). Such mutations might be expected to impair the functional efficiency of respiratory chains. There is recent evidence that mitochondrial superoxide production is notably high in melanomas that have developed resistance to the BRAF inhibitor vemurafenib (14); hence, such cancers may be good candidates for i.v. ascorbate therapy.

A corollary of these considerations is that an increase in substrate delivery to cancer mitochondria would be expected to boost superoxide production – and hence sensitize cancer cells to concurrent sodium ascorbate therapy.

A parallel line of research has demonstrated that superoxide production by NADPH oxidase complexes is elevated in a high proportion of cancer cell lines (7, 15). It appears that NADPH oxidase activation and dysfunctional mitochondria collaborate to increase superoxide production in a high proportion of cancers. Arguably, this phenomenon may contribute to the selective sensitivity of malignant cell lines to high concentrations of extracellular ascorbate *in vitro*. Whether Nox2 activity in the plasma membrane is of importance in this regard might be questioned, as this generates superoxide extracellularly. (The possibility that extracellular production of hydroxyl radical in the microenvironment of Nox2 complexes might induce membrane damage might however be entertained.) However, other forms of Nox are expressed intracellularly, and Nox4 is often found in association with nuclear membranes, where it can promote oxidative damage to DNA (16, 17). As noted, both apocynin and Nox4 anti-sense treatment markedly alleviated the toxicity of extracellular ascorbate to mesothelioma cells (8).

Since superoxide does not readily traverse membranes, the subcellular compartment in which superoxide is generated should determine the site of hydroxyl radical generation when cancer cells are exposed to extracellular ascorbate. The fact that ascorbate-mediated cell death shows features of both necrosis and apoptosis suggests that damage to DNA is not the sole mediator of this toxicity, though it might play a role in this regard (3).

A moderate increase in superoxide production within cancers may in fact be selected for, as an increase in hydrogen peroxide level can reversibly inhibit tyrosine phosphatase activities that target tyrosine kinases promoting cancer malignancy and survival (18, 19). Hydrogen peroxide can also boost the activity of another pro-malignancy factor, hypoxia-inducible factor-1 (HIF-1), by oxidizing intracellular ascorbate and thereby inhibiting proline and asparagyl hydroxylases that target it for proteasomal degradation and inhibit the function of its transactivational domain (20–24). And activation of NF-kappaB, a mediator of aggressive behavior and chemoresistance in many cancers, can also be up-regulated by hydrogen peroxide (25). Moreover, oxidative stress in cancers or pre-neoplastic tissues promotes the genetic lability that promotes cancer induction, progression, and chemoresistance (26). The relative deficit of catalase activity in some cancers – which presumably renders the hydrogen peroxide generated during i.v. ascorbate therapy more effective – likewise may be selected for.

The high constitutive activity of HIF-1 in many aggressive cancers – reflecting tumor hypoxia as well as increases in growth factor and NF-kappaB signaling (27) – is now believed to be a key mediator of the “Warburg effect,” wherein cancers are characterized by an up-regulation of aerobic glycolysis and decrease in mitochondrial respiratory activity (28, 29). This increase in HIF-1 activity works to lessen oxidative stress in tumors in various ways. It does so in part via induction of pyruvate dehydrogenase kinase (PDK), an inhibitor of the pyruvate dehydrogenase complex (30–32). When pyruvate dehydrogenase is inhibited, glycolytically generated pyruvate is more likely to be converted to lactate and secreted, than it is to serve as substrate for mitochondrial oxidation. As a result, mitochondrial superoxide generation is decreased. Moreover, HIF-1’s inductive effect on glucose transporters, as well as key enzymes of the glycolytic pathway and hexose phosphate shunt, provides antioxidant protection to cancers (33). The hexose phosphate shunt generates the NADPH needed to maintain glutathione in its reduced configuration; reduced glutathione is of crucial importance to the antioxidant defenses of cells. And the pyruvate generated by glycolysis can directly quench peroxides (34, 35). [Conversely, glucose deprivation or 2-deoxyglucose administration can impair cancer’s antioxidant defenses (33)]. Hence, HIF-1 activity tends to decrease superoxide production while boosting the antioxidant defenses of cancer cells. Recently, Sinnberg and colleagues have demonstrated that exposure of cancer cells lines to the HIF-1 activator cobalt, or to severe hypoxia, can greatly lessen the sensitivity of cancer cells lines to killing by extracellular ascorbate (36). They propose that this phenomenon may explain why published clinical trials with i.v. ascorbate therapy have failed to report objective responses, in sharp contrast to the lethal impact of low millimolar ascorbate on cancer cell lines *in vitro*. Of course, severe hypoxia could also be expected to lessen ascorbate’s ability to generate extracellular superoxide (7).

## Increasing Substrate Delivery to Cancer Mitochondria – Dichloroacetate and Ketosis

It follows that strategies that suppress HIF-1 activity in cancers would likely be useful as adjuvants to i.v. ascorbate therapy – provided that these strategies do not hinge on boosting antioxidant activity in the tumor. mTORC1 inhibitors (e.g., rapalogs and metformin), salicylate, and high-dose alpha-ketoglutarate may have some utility in this regard, as reviewed recently (27). A particularly interesting and quite feasible option would be to administer dichloroacetate (DCA), a PDK inhibitor, which disinhibits pyruvate dehydrogenase activity. By routing more pyruvate to mitochondrial oxidation, DCA could be expected to enhance mitochondrial superoxide production, while decreasing the pool of pyruvate that functions as a direct oxidant scavenger (37–40). Indeed, Kluza and colleagues have recently reported that DCA increases mitochondrial superoxide production in a melanoma cell line, and potentiates the cytotoxic activity of elesclomol, a drug-in-development, which acts on mitochondria to potentiate their superoxide production (41). DCA has the particular merit that it can be expected to increase mitochondrial substrate availability more notably in cancer cells characterized by HIF-1 activation (i.e., which express the Warburg phenotype) than in most healthy cells, owing to the fact that such cancer cells tend to have much higher PDK activity than normal cells. DCA has the potential to decrease HIF-1 activity by breaking a positive feedback loop whereby the build-up of glycolytic intermediates secondary to pyruvate dehydrogenase inhibition stabilizes HIF-1 (42). It should be noted that oral DCA in amounts sufficient to inhibit PDK activity *in vivo* is well tolerated, aside from mild distal paresthesias seen in a minority of patients during prolonged treatment (43, 44); hence, it likely would be well tolerated if given episodically in conjunction with i.v. ascorbate therapy.

An alternative means of boosting substrate delivery to cancer mitochondria – while modestly lessening the ability of glucose to provide antioxidant protection to cancers – is to feed a ketogenic diet. In mice bearing human lung cancer xenografts, a ketogenic diet has been shown to increase oxidative stress within the tumors, and sensitize these tumors to concurrent radiotherapy (45). Evidently, under ketotic conditions, cancers will derive a higher proportion of their ATP from the oxidation of free fatty acids and ketone bodies, which are mitochondrial substrates.

## Coping with Tumor Hypoxia

This strategy of amplifying mitochondrial activity within cancers as an adjuvant to i.v. ascorbate has a potential drawback – by increasing tumor oxygen consumption, it could be expected to amplify tumor hypoxia. Indeed, Zwicker et al. report that, whereas, DCA enhances the radiosensitivity of human cancer cell lines *in vitro*, it actually lessens their radiosensitivity *in vivo*, apparently because DCA treatment enhances tumor hypoxia (46). Since intense hypoxia could be expected to compromise ascorbate’s ability to generate superoxide, it is conceivable that this phenomenon would be pertinent to i.v. ascorbate therapy. Conversely, measures which boost tumor oxygen levels may have potential for increasing the efficacy of such therapy (36). Infusion of perfluorochemical oxygen carriers – used safely as “artificial blood” – has the ability to alleviate tumor hypoxia to some degree. The perfluorochemical agent perftoran, approved for clinical use in Russia and Mexico, has been employed at Oasis of Hope Hospital as an adjuvant to its i.v. ascorbate cancer protocol (47). Hyperbaric oxygen, which can be employed as a radiosensitizer, might also be worth exploring in the context of ascorbate therapy (48, 49).

## Hyperthermia Boosts Superoxide Production in Cancers

An additional strategy for boosting superoxide production within cancer cells is hyperthermia. A number of studies have reported that cancer cells incubated at 42–43°C experience increased oxidative stress, and that antioxidant measures render this heat stress less cytocidal (50–54). Notably, transfection of cancer cells with the mitochondrial superoxide dismutase markedly lessens their sensitivity to heat killing (53, 55). This strongly suggests that increased mitochondrial generation of superoxide is largely responsible for the oxidative stress imposed by hyperthermia on cancer cells. Conceivably, the somewhat selective toxicity of hyperthermia to cancer cells may reflect their tendency to express dysfunctional mitochondria with an increased propensity to generate superoxide.

Of particular interest is a study showing that heat increases the cytotoxicity of hydrogen peroxide to Chinese hamster ovary cells (56). Moreover, in nude mice peritoneally seeded with a human colorectal cancer, hyperthermic intraperitoneal perfusion slowed the growth of the cancer, and co-infusion of tolerable levels of hydrogen peroxide greatly amplified the response seen with hyperthermia alone (57). Since i.v. ascorbate therapy works by generating hydrogen peroxide within the extracellular space of tumors, these findings predict that concurrent hyperthermia treatment (achieving temperatures at or near 42°C) should potentiate the therapeutic response to i.v. ascorbate – or vice versa.

## Mitochondrial Toxins

Several agents, including the investigational drug elesclomol and aqueous extracts of the Chinese medicinal herb *Scutellaria barbata*, have been reported to somewhat selectively enhance mitochondrial oxidative stress in cancer cells (58–62). The molecular target(s) and the basis for the cancer-selectivity of these agents is not yet clear. Elesclomol’s efficacy appears to hinge on its ability to chelate copper ions and transport them into mitochondria, where they potentiate mitochondrial oxidant stress. Since these drugs are not known to inhibit PDK, they potentially could synergize with DCA in promoting mitochondrially generated oxidative stress. Indeed, exposure to DCA, or knock-down of PDK3 with siRNA, has been shown to potentiate elesclomol’s induction of oxidative stress in melanoma cell lines, and concurrent treatment with DCA amplified the growth retardation of a human melanoma achieved with elesclomol in nude mice (41). Not surprisingly, elesclomol’s clinical efficacy in melanoma appears to be greater in patients whose serum LDH levels are not increased; an elevated serum LDH in melanoma patients is presumed to reflect the presence of cancer with a high Warburg phenotype, in which mitochondrial respiratory activity is low (63). [LDH is HIF-1 inducible, and hence its expression is increased in this phenotype (64)]. It would be interesting to observe the interaction of these strategies with intravenous ascorbate. The elesclomol–copper complex is capable of oxidizing ascorbate to generate hydrogen peroxide (65); it therefore might potentiate ascorbate’s ability to generate hydrogen peroxide within the extracellular space. Importantly, the reduced elesclomol–copper complex does not interact with hydrogen peroxide to generate hydroxyl radical, so it may be safe for use with i.v. ascorbate (65). Menadione, which likewise can catalyze the transfer of electrons from ascorbate to oxygen, potentiates the efficacy of ascorbate therapy in rodent cancer models, without notable adverse effects (66, 67).

## Labile Iron Pool as a Determinant of Response to Intravenous Ascorbate Therapy

The Haber–Weiss reaction requires not only superoxide and hydrogen peroxide, but also free labile iron or copper. The labile iron pool (LIP) of cancer cells may therefore be another determinant of a cancer’s sensitivity to intravenous ascorbate therapy; this is consistent with a report that an intracellular but not extracellular iron chelator alleviates the toxicity of high extracellular ascorbate to cancer cell lines (6). It has been proposed that the selective susceptibility of many cancers to the cytotoxic effects of the anti-malarial drugs artemisinin or dihydroartemisinin may reflect increased amounts of free labile iron in those cancers, which interact with artemisinin to generate toxic radicals (68–70). Although relatively few studies have compared LIP levels in cancers and healthy tissues, one recent study found that LIP was consistently higher in breast cancer cells lines than in non-transformed breast epithelial cell lines (71). Iron deficiency or chelation tends to slow cancer growth (72–74). Conversely, genetic expression patterns that predict efficient iron uptake (high transferrin receptor, low hereditary hemochromatosis), and low capacity for iron export (low transportin, high hepcidin) are associated with greater risk of recurrence and decreased survival in breast cancer patients (71, 75). Elevated c-Myc activity, a driver of transformation in many cancers, tends to increase the LIP by promoting transcription of the transferrin receptor and IRP2, while suppressing transcription of the ferritin heavy chain (FHC) (76–79). HIF-1 activity is constitutively active in many aggressive cancers, and is typically elevated in hypoxic tumor regions (80, 81); since HIF-1 promotes transcription of the gene coding for the transferrin receptor, it would be expected to increase the intracellular iron pool (82–85). This predicts that LIP should be relatively high in many aggressive cancers. Moreover, the fact that ample iron availability supports rapid cancer growth and spread, suggests that cancer variants with high LIP will tend to be selected for as cancers progress. Hence, there is reason to suspect that LIP is elevated in many cancers, particularly those that are advanced and aggressive.

## Inhibiting NF-kappaB Activity with Salicylate May Increase the LIP in Cancers

On the other hand, too high an LIP can subject cancers to cytotoxic levels of oxidative stress. The constitutive activation of NF-kappaB characteristic of many cancers provides protection in this regard. NF-kappaB promotes transcription of the gene for FHC, counteracting the influence of c-Myc in this regard (86–88). FHC is not only crucial for iron sequestration, but also possesses a ferroxidase activity that converts toxic Fe^+2^ to more benign Fe^+3^, contributing importantly to antioxidant protection (89). Hence, measures which inhibit constitutive NF-kappaB activity in cancers tend to increase the LIP and promote oxidative stress, as has been reported (87, 88). In contrast, the absence of constitutive NF-kappaB activity in healthy cells implies that NF-kappaB inhibitors will have little impact on their FHC levels, which tend to be adequate since c-Myc is not over-expressed.

Although many chemical agents have been developed capable of suppressing NF-kappaB activation, the one which is clinically available is the naturally occurring agent salicylate (also available as salsalate, a better-tolerated dimer of salicylate that functions as a pro-drug). Salicylate down-regulates NF-kappaB activity in cancers by inhibiting IkappaB kinase-beta, a central mediator of NF-kappaB activation (90, 91). A recent report that dihydroartemisinin and salicylate have a complementary impact on killing of Molt-4 leukemia cells, likely reflects the fact that salicylate, via inhibition of NF-kappaB activity, decreases ferritin expression and hence increases the LIP, sensitizing the cells to killing by the dihydroartemisinin (92). [Up-regulation of the apoptotic response to dihydroartemisinin, and retention of this drug in cancer cells owing to decreased expression of the multidrug resistance protein, are additional possibilities in this regard (91, 93)]. Other factors being equal, it is reasonable to predict that pre-administration of salicylate, by increasing the LIP in cancer cells, would also sensitize cancer to destruction by intravenous ascorbate therapy. This drug, as well as its pro-drug salsalate, has been used for decades in the treatment of rheumatoid arthritis. These agents do not produce the gastrointestinal bleeding or renal damage seen with NSAIDS (as their impact on cyclooxygenase activity is very mild and transient); their dose-limiting side effect is ototoxicity, which is fully and rapidly reversible (94–96).

Another approach to increasing LIP in cancer cells prior to i.v. ascorbate therapy would be to iron load, orally or via intravenous iron administration. This would be expected to have a somewhat selective impact on the LIP of the many cancers in which the transferrin receptor is highly expressed. High oral daily doses of ferrous sulfate were reported to enhance the sensitivity of a rat fibrosarcoma to concurrent treatment with dihydroartemisinin; *in vitro*, concurrent exposure to holotransferrin boosted the sensitivity of breast cancer cells to this drug (97, 98). Clinically, intravenous administration of iron might prove to be a quicker and better-tolerated strategy for achieving an acute increase in the LIP in cancers (99).

## Potentiating the Lethality of Hydroxyl Radicals with PARP Inhibition

Since generation of hydroxyl radicals in the microvicinity of DNA gives rise to many types of DNA damage (100, 101), it stands to reason that tolerable concurrent measures which impede the efficiency of DNA repair should potentiate the chance that such damage will prove lethal to the cell. Poly(adenosine diphosphate-ribose) polymerase (PARP) plays a key role in the repair of single strand breaks that arise after oxidative damage to DNA (102), and clinical PARP inhibitors are currently being evaluated clinically in cancer, either used alone or in conjunction with cytotoxic therapies; these drugs are reasonably well tolerated in doses that can suppress PARP activity *in vivo* (103, 104). Ma and colleagues have recently reported that concurrent exposure of an ovarian cancer cell line to the PARP inhibitor olaparib (20 μM, 24 h) markedly potentiated the cell kill achieved with ascorbate (2.5 mM, 24 h); whereas, about 60% of the cells survived exposure to ascorbate alone, only about 15% of the cells survived ascorbate plus olaparib (105). Hence, PARP inhibitors may have important potential as adjuvants to i.v. ascorbate therapy. This finding also suggests that nuclear generation of hydroxyl radical is a mediator of the cytotoxicity of extracellular ascorbate to cancer cells.

Ironically, it is still unclear whether PARP inhibitors will achieve FDA approval, as the survival benefits and safety of these drugs in recent clinical studies have not been clear cut (106–110). This may reflect the fact that PARP inhibitors can potentiate the damaging impact of cytotoxic chemotherapy on normal tissues, and, when used alone, spontaneous damage to tumor DNA may be too modest for these agents to achieve an important impact (except possibly in patients carrying germline mutations in BRCA proteins, required for repair of DNA double-strand breaks). In contrast, since severe oxidative stress during i.v. ascorbate therapy appears to be confined to the cancer, PARP inhibitors may prove to be quite safe for use in conjunction with such therapy. Hence, once the capacity of i.v. ascorbate therapy to generate hydroxyl radicals selectively in cancers has been optimized, concurrent PARP inhibition may have important potential for safely enhancing its efficacy.

## In Overview

**Figure 1:**
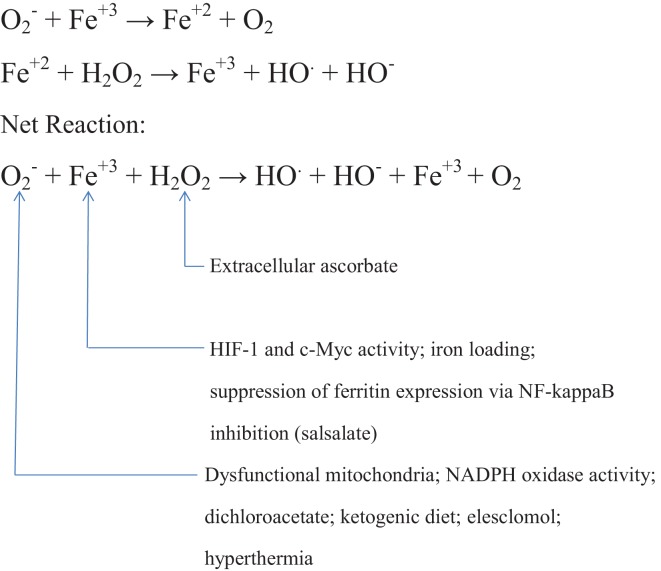
**Postulated role of the Haber–Weiss reaction in mediating the toxicity of high extracellular ascorbate to cancer cells, with strategies for potentiating this reaction**.

Figure 1 provides a summary of some of the facts and strategies cited above.

The fact that a high proportion of cancers generate increased amounts of superoxide, largely via somewhat dysfunctional mitochondria and/or active NADPH oxidase complexes, provides a straightforward and satisfying rationale for the fact that cancer cells are selectively susceptible to killing by hydrogen peroxide and millimolar concentrations of ascorbate; metal-catalyzed interaction of tumor-produced superoxide with the hydrogen peroxide generated extracellularly by high levels of ascorbate will generate hydroxyl radicals within the cell. It follows that i.v. ascorbate therapy should be more effective if adjuvant strategies are concurrently employed that boost superoxide production somewhat selectively in cancers. One approach to achieve this is to provide additional substrate for mitochondrial oxidation. In the high proportion of cancers that express the Warburg effect – reflecting in part constitutive activation of HIF-1 – inhibition of HIF-1-inducible PDK with DCA increases mitochondrial oxidation of pyruvate. Alternatively, the elevations of free fatty acids and ketone bodies associated with ketotic diets – coupled with a modest reduction in the glucose needed for glycolytic ATP generation – could also be expected to boost mitochondrial respiration in cancer cells. Measures which target HIF-1 expression, such as salsalate and mTORC1 inhibitors, should also boost mitochondrial respiration while suppressing the antioxidant effects of cancer glucose metabolism. Hyperthermia near 42°C promotes oxidative stress in cancer cells – most likely by boosting mitochondrial superoxide generation – and several studies show that hydrogen peroxide and hyperthermia can synergize to kill cancer cells. The copper-transporting investigational drug elesclomol promotes oxidative stress in mitochondria – presumably by copper catalysis – and this effect is amplified by the increased mitochondrial respiration provoked by DCA in cancers with the Warburg phenotype. The interaction of elesclomol with exogenous hydrogen peroxide in cancers has not yet been studied, but should be. Like menadione, elesclomol may potentiate ascorbate’s ability to generate extracellular hydrogen peroxide. Tumor hypoxia renders cancer cells more difficult to kill with ascorbate than are well-aerated cancer cells in culture; HIF-1 activation as well as a decrease in oxygen availability for interaction with ascorbate play a role in this. Measures which increase tumor respiration will have the countervailing negative effect of potentiating tumor hypoxia; hence, concurrent tumor oxygenation using perfluorochemical oxygen carriers or hyperbaric oxygen may improve responses to i.v. ascorbate therapy. Increasing the cancer pool of labile iron by antagonizing NF-kappaB activity with salicylate, and by intravenous iron administration, are additional strategies that might potentiate the efficacy of i.v. ascorbate therapy of cancer. And concurrent administration of PARP inhibitors has potential for amplifying the lethality of oxidant-induced DNA damage.

Despite encouraging anecdotal reports and promising results in xenograft models (5, 6, 111, 112), the initial formal phase I clinical trials of intravenous high-dose ascorbate therapy for cancer have so far yielded little evidence of objective response (2, 113). The possibility that ascorbate therapy can improve the response to concurrent chemotherapies, as suggested in rodent studies, is now under active investigation (6, 105, 114, 115). If i.v. ascorbate therapy *per se* is to evolve into an important tool for cancer control, it appears that adjuvant strategies that complement its impact on oxidative stress will be required. Complex strategies, which combine several of the therapeutic options cited above in a robust “oxidative stress therapy of cancer” (116, 117) may have important potential for cancer control, and could be evaluated first in xenograft models.

The mechanistic speculations offered here are based largely on the results of cell culture studies, which may limit their pertinence to the impact of high-dose ascorbate *in vivo*. Nonetheless, the utility of parenteral ascorbate has been well documented in rodent tumor models.

It should also be noted that measures which notably enhance intracellular ascorbate levels in cancers – such as correction of overt vitamin C deficiency, or parenteral administration of dehydroascorbic acid – have the potential to slow tumor growth and boost chemosensitivity by promoting proteasomal degradation of hypoxia factor-1, a mediator of aggressive growth and chemoresistance in many cancers (23, 118–121). This phenomenon would not be expected to induce the cancer cell death seen when cancer cells are exposed to high extracellular concentrations of vitamin C. However, if parenteral ascorbate can increase the intracellular ascorbate levels of tumors in normally nourished mice – an issue which requires clarification – this effect might contribute to the retardation of cancer growth observed in rodent studies with parenteral ascorbate. The failure of *oral* vitamin C to control the growth of advanced cancers in controlled clinical trials may reflect the facts that such a regimen can only modestly and transiently enhance plasma levels of ascorbate, and that plasma levels of ascorbate in reasonably well nourished subjects are sufficient to saturate the activity of membrane ascorbate transporters (121, 122).

## Conflict of Interest Statement

Oasis of Hope Hospital practices i.v. ascorbate therapy of cancer, but has no proprietary ownership of any of the strategies discussed here.
